# EspA-Intimin chimeric protein, a candidate vaccine against *Escherichia coli* O157:H7

**Published:** 2013-09

**Authors:** Hamid Sedighian Rad, Seyed Latif Mousavi, Iraj Rasooli, Jafar Amani, Moohamad Reza Jalali Nadooshan

**Affiliations:** 1 Applied Microbiology Research Center, Baqiyatallah Medical Science University, Tehran; 2 Department of Biology, Faculty of Basic Sciences, Shahed University, Tehran, Iran; 3 Department of Pathology, Faculty of Medical Sciences, Shahed University, Tehran, Iran

**Keywords:** Enterohemorrhagic *Escherichia coli*, Chimeric vaccine, Intimin, EspA

## Abstract

**Background and Objective:**

Enterohemorrhagic *Escherichia coli* (EHEC) O157:H7 is an important enteric pathogen in human causing bloody or nonbloody diarrhea, which may be complicated by hemolytic uremic syndrome (HUS). Cattle are an important reservoir of EHEC. This research aims at vaccination with a divalent chimer protein composed of EspA_120_ and Intimin 282 and its preventive effect of EHEC O157 colonization in mice rectal epithelium.

**Materials and Methods:**

A divalent recombinant EspA-Intimin (EI) protein containing EspA_120_ and Intimin_280_ attached with a linker was amplified from a trivalent construct and cloned in pET-28a (+) vector. The immunization was conducted in mice after expression and purification of the recombinant EI (rEI).

**Results:**

Mice subcutaneously immunized with rEI, elicited significant rEI specific serum IgG antibodies and showed significantly decreased *E.coli* O157:H7 shedding compared to the control group.

**Conclusion:**

The chimeric recombinant protein induced strong humoral response as well as protection against oral challenges with live *E.coli* O157:H7.

## INTRODUCTION

Enterohemorrhagic *Escherichia coli* (EHEC) is one of the most important food and waterborne zoonotic pathogens causing hemorrhagic colitis which can lead to the hemolytic-uremic syndrome (HUS) in human. The predominant serotype of EHEC is O157:H7 and the most important reservoir are cattle (1, 2). EHEC in many cases is similar to *Shigella dysenteriae* which produces toxins, known as verotoxins or Shiga-like toxins (stx) (3).

EHEC belongs to a family of pathogens causing attaching and effacing (A/E) lesions (4). EHEC and the closely related pathogen enteropathogenic *Escherichia coli* (EPEC) possess a homologous chromosomal DNA region called the Locus of Enterocyte Effacement (LEE) that contains all the genes required for A/E lesion formation (5).

The LEE contains three major regions with known functions. One of the LEE regions encodes several proteins that are secreted via the type III secretion system (TTSS) which delivers these factors directly into the host cells. These factors include EspA, EspB and EspD (5, 6). All of three factors mentioned above, are essential for signal transduction in mammalian host cells and also for A/E lesion formation. EspA is a protein with structural role and is believed to be the major component of a large filamentous organelle that has a transient expression on the bacterial surface and delivers EspB and EspD directly to the host cell membrane (7, 8). During the early stage of A/E lesion formation, this protein is found to interact with the host cell and also is involved in forming a bridge between the epithelial cells and surface of the bacteria at this stage. Through this bridge, Tir protein is transferred into the host cell and acts as a receptor for an integral outer membrane protein of EHEC called Intimin (9, 10). Intimin protein encoded by *eae* gene and it is essential for attachment of the bacterium to the surface of host cell and leads to the disruption of cytoskeleton regulating network (11). Biophysical and Biochemical studies on the Intimin from different strains have shown that Intimin can be subdivided into three regions (flexible N-terminal region, central membrane-integral β-barrel and surface- exposed C-terminal region). C-terminal region resides includes four extracellular domains named D0 to D3, where the receptor-binding activity resides and it's located on 280 C-terminal amino acid region (int280) (12). Phylogenies and aserological examination have recognized at least six different intimin subtypes designated Int- α, β, γ, δ, ε and θ, that differ in the sequence of the carboxy-terminal cell-binding domain (13). The outer membrane proteins such as Intimin and TTSS proteins like EspA, are the most important factors contributing to EHEC O157:H7 colonization. There are currently no available vaccines to prevent disease resulted by EHEC, but a number of trial approaches are being investigated in animals (14). Vaccination with the C-terminal of intimin, induced strong response of specific antibodies in serum and colostrums of pregnant swine (15) and reduced the time of *E. coli* O157:H7 shedding in mice (16). Vaccination of cattle with a combination of EspA, intimin-531 and Tir significantly reduced the total levels of EHEC O157 (17). Vaccination of mice with a trivalent protein containing EspA, Intimin was is a successful method to reduce shedding levels of *E. coli* O157:H7 following oral challenge (18).

In this research we assayed vaccination with a divalent chimer protein composed of EspA_120_ (lacking 36 amino acids from the N-terminal) and Intimin _282_ (282 amino acids from the C-terminal) and its preventive effect of EHEC O157 colonization in mice rectal epithelium.

## MATERIALS AND METHODS

### Plasmids and Bacterial strains

The plasmids and bacterial strains used in this research are pET28a, DH5α and BL21DE3 (Pasteur Institute of Iran). *E. coli* strains were grown in Luria-Bertani (Merck) broth at 37°C. Kanamycin (40µg/ml, Sigma) was added to freshly autoclaved solid and broth medium.

### Amplification of synthetic gene

The gene encoding a divalent chimeric protein containing EspA_120_ and Intimin _280_ with spacer linker (EAAAK) 4 was obtained via PCR from a reference trivalent synthetic gene, GenBank accession number FJ744505 (19) (Fig. 4). The two primers used in this study, were 5'-ATATAGGATCCGCGGATATGAACG-3' with *Bam*HI and 5'-TACTATAAGCTTTTATTCCACGCACACC-3' with *Hin*dIII restriction sites as forward and reverse primers respectively (ShineGene).

### Cloning and expression of the recombinant fragment

PCR reaction was performed in a final volume of 25 µl containing 1 x PCR buffer, 0.2 mM dNTPs mix, 4 mM MgSo4, 2 pM of each primer and 5 U *Pfu* DNA polymerase (Fermentas). PCR was performed as: initial denaturation at 94°C for 8 min, denaturation at 94°C for 1 min, annealing at 58°C for 30 sec and extension at 72°C for 90 s(35 cycles) with the final extension at 72°C for 10 min. The PCR product was digested with the restriction enzymes *Bam*HI and *Hin*dIII (Fermentas), cloned in pET28a expression vector and transformed into *E.coli* strain BL21 (DE3) by elcteroporation (Bio Rad). The recombinant clones were analyzed using restriction enzymes *Bam*HI and *Hin*dIII confirmed by sequencing. A single colony of *E.coli* BL21 (DE3) containing pET-EI recombinant plasmid was picked up and grown in 5 ml LB broth overnight at 37°C. 5ml of the overnight culture was transferred into 500 ml LB medium with 40µg/ml kanamycin and was grown at 37°C to an optical density (600 nm) of 0.8. Expression of the protein was induced with 1 mM IPTG (Sigma) and the cells were grown for 6 h. Cells were harvested by centrifugation at 3500 g for 5 min and pellets were suspended in 1 ml of lysis buffer (50 mM NaH_2_PO_4_ pH 8.0, 300 mM NaCl , 0.2 mg/ml lysozyme (20 mg/ml SinaColon). The samples were analyzed for the expression of protein by SDS-PAGE.

### Purification of recombinant protein

The recombinant protein was purified by Ni–NTA affinity chromatography (Qiagen). The freeze cell pellet was thawed for 15 min on ice and resuspended in lysis buffer at 5 ml per gram wet weight and was then incubated on ice for 30 min. The lysate was then sonicated (6 times, 10 s at 200 W with a 10 s cooling period between each burst) using a sonicator equipped with a micro tip. The lysate was then centrifuged at 1500×g for 20 min at 4°C to precipitate the cellular debris. The supernatant was loaded onto a Ni–NTA agarose column, pre equilibrated with 5ml lysis buffer containing 10 mM imidazole. The protein was eluted by a stepwise procedure, using buffers containing 40, 100 and 200 mM imidazole followed by 1ml of 20 mM MES (2-N-morpholinoethanesulfonic acid) buffer. The purified chimeric protein (EI) was monitored on 12% SDS-PAGE. The purified protein was dialyzed against Tris (50 mM) and protein concentration was estimated by the Bradford method.

### Western blot analysis

The purified protein (EI) was transferred to nitrocellulose membrane using transfer buffer (39 mM glycine, 48 mM Tris-base, 0.037% SDS, and 20% methanol, Bio-Rad). The membrane was incubated in the blocking buffer containing 5% bovine serum albumin (BSA) with gentle shaking at 4°C overnight. The membrane was incubated in a 1:3000 dilution of mice anti-His-tag specific antibody in the PBS containing 0.05% Tween 20 (PBS/T), with gentle shaking for 1 h at room temperature. The membrane was washed with PBS/T three times, each time for 5 min and was then incubated in 1:1000 dilution of HRP-conjugated goat antimouse IgG antibody (Sigma), with gentle shaking for 1 h at room temperature. After washing, the membrane with PBS/T for three times, detection was carried out using BM Blue pod precipitation (Roche). Chromogenic reaction was stopped by rinsing the membrane twice with distilled water and drying on filter paper.

### Animal immunization

Five week old BALB/c mice used in this study were obtained from the Pasteur Institute of Iran and animals were housed and treated incompliance with regulations of the International Council on Animal Care. Mice were divided into test and control groups. In the test group, each mouse was injected subcutaneously in the back of neck with 20µg recombinant protein (EI) with complete Freund's adjuvant (Sigma). 15µg,10µg of rEI protein was injected as a first, second and third booster after 20 and 35 days, using incomplete Freund's adjuvant. Animals received 5µg of rEI protein intraperitoneally as the last booster 50 days after the first injection (Table 1). The control group was injected with sterile PBS following the same protocol. Blood samples were collected from the mice a week after each booster dose.

**Table 1:** Summary of immunization protocols

Group	n	Initial Immunization		Booster 1		Booster 2		Booster 3	

		components	Quantity (µl)	components	Quantity (µl)	components	Quantity (µl)	components	Quantity (µl)
control	4	PBS	100	PBS	100	PBS	100	PBS	100
		C.F.A*	100	I.F.A**	100	I.F.A	100	I.F.A	100
rEI	7	rEI	(20 µg)	rEI	(15 µg)	rEI	(10 µg)	rEI	(5 µg)
		C.F.A	100	I.F.A	100	I.F.A	100	I.F.A	100

* C.F.A= Complete Freund's Adjuvant

** I.F.A= Incomplete Freund's Adjuvant

### Quantification of specific humoral antibody

Antigen-specific antibody responses were determined by an enzyme linked immunosorbent assay (ELISA). Polystyrene 96-well plates (Nunc) were coated with 5 µg of rEI diluted in 100 µl coating buffer (64 mM Na_2_CO_3_, 136 mM NaHCO_3_, pH 9.8) overnight at 4C. The plates were washed three times with PBS/T. Nonspecific binding sites were blocked with 100 µl of 5% dry milk in PBS. Mouse serum samples were serially diluted to 1:500 in phosphate-buffered saline containing 0.05% Tween 20 and were added to the ELISA plates. The plates were incubated for 1h at 37C and were then washed three times in PBST. 100µl of O-phenylenediaminedihydrochloride (OPD) (Sigma) was added to each well and incubated at room temperature for 15 min. The reaction was stopped with 100 µl of 2 M H_2_SO_4_ and the OD_492_ was read on a microplate reader (Bio-Rad).

### Challenging the immunized mice

The mice were challenged 14 days after the last immunization. In order to reduce the normal flora of the gut, each mouse in both test and control groups was given drinking water containing streptomycin sulfate (5 mg/ml). After 24 h of treatment mice were fasted overnight, and then were fed with 10^10^ CFU of live *E. coli* O157:H7 (ATCC: 35218) suspended in 100µl of sterile PBS. The fecal samples from the mice were collected at two days interval for 14 days. Shedding of *E.coli* O157:H7 in fecal samples was monitored by adding approximately 0.1 g of feces to 1ml LB broth. The mixture was incubated at 37°C for three hours to allow fecal pellets to soften. The mixture was vortexed until pellets were no longer visible. Serially diluted samples from supernatant were plated on Sorbitol MacConkey agar containing cefixime and tellurite. Plates were incubated overnight at 37°C and *E. coli* O157:H7 colonies were counted and bacterial colonies were tested for the O157 antigen by latex agglutination (20, 21).

### Histological examination

Eight days after observing the first signs of disease, 4 mice from test and 3 mice from control groups were selected and sacrificed. Colons and cecums were removed and rinsed thoroughly in sterile PBS and fixed in 10% formalin for microscopic examination. Formalin-fixed tissues were processed, paraffin embedded sectioned at 5 µm, and stained with haematoxylin. Sections were examined by light microscopy, for the presence of adhering *E.coli* O157:H7 on intestine cells, The bacterial count was average of 100 microscopic fields (22).

### Statistical analysis

Statistical analyses was performed using a SPSS 13.0 statistical program. The data in each figure was a representative of three independent experiments expressed as the mean ± standard deviation (SD). Student t-test was also used to evaluate the data for antibody responses between immunized and non-immunized groups; also t-test was used to analyze the data from fecal shedding of bacteria. A probability level of *p*<0.05 was considered significant.

## RESULTS

### Amplification and cloning of EI gene

**Fig. 1:**
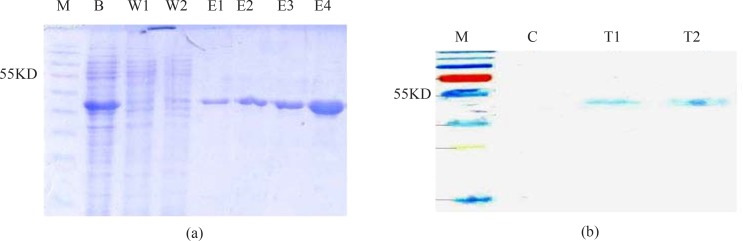
PCR products and digestion analysis on agarose gel. a) Lane M, DNA size marker; Lane *EI*, *espA-intimin gene*. b) Lane M, DNA size marker 1kb; Lanes 1-3, digested constructs of by *Bam*HI and *Hin*dIII restriction enzymes.

*ei* gene was amplified by PCR using specific primers. The PCR product (1290 bp) is shown in Fig. 1a. The fragment was cloned in pET-28a (+) vector and then transformed into *E. coli* BL21DE3. pET28a-EI plasmids were extracted from *E.coli* and digested by *Bam*HI/*Hin*dIII and analyzed by agarose gel electrophoresis (Fig. 1b).

### Expression and purification of chimeric recombinant protein

**Fig. 2:**
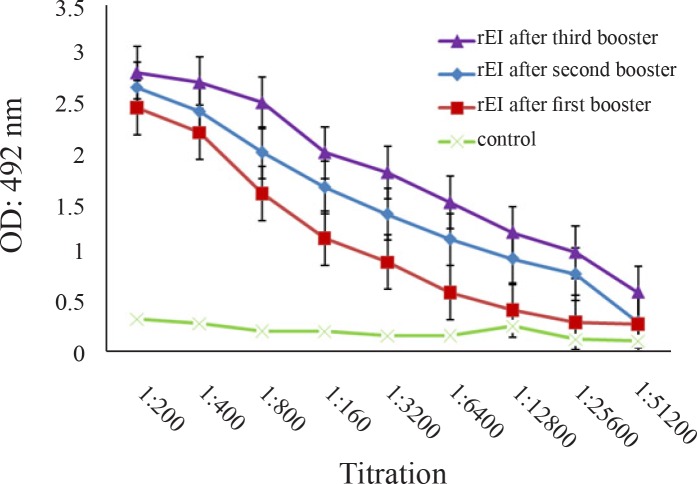
Purification and identification of rEI. a) Purification steps of rEI. M, protein size marker. B, cleared lysate before passing through column; W1 &W2, Flow through; E1-4, elution with 250 mM imidazole. b) Western blotting of rEI. M, protein size marker; C, non-induced transformed (pET28a without insert) BL21DE3 as control; T1, supernatant of induced transformed BL21DE3; T2, purified chimeric recombinant protein.

Recombinant EI protein with N-terminal His tag was expressed in *E. coli* BL21DE3 and purified by Ni-NTA affinity chromatography. The SDS-PAGE analysis of the purified product is demonstrated in Fig. 2a. There is no extra band in line E4 of the figure indicating high purity of the protein. Protein concentration was estimated by the Bradford protein assay and the yield of EI protein was 50 mg/L culture. Western blot analysis with the anti-His-tag antibodies was confirmed the presence of chimeric proteins with a size of 46 kDa (Fig. 2b).

### Immune response elicited with recombinant EI (rEI) protein

**Fig. 3:**
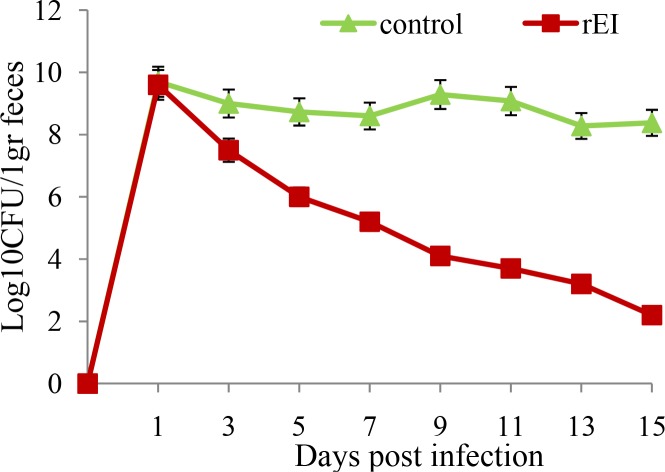
Serum antibody response of BALB/c mice immunized with recombinant EI proteins.

The pattern of antibody production followed by immunization at three settings of first, second and third injections were the same except for increased antibody titer after each immunization (Fig. 3). Results from subcutaneously immunized animals with purified rEI protein in the test group showed significant (*p* < 0.05) EI-specific IgG antibodies up to 1/512000 dilution in comparison with the control group (Fig. 3).

**Fig. 4:**
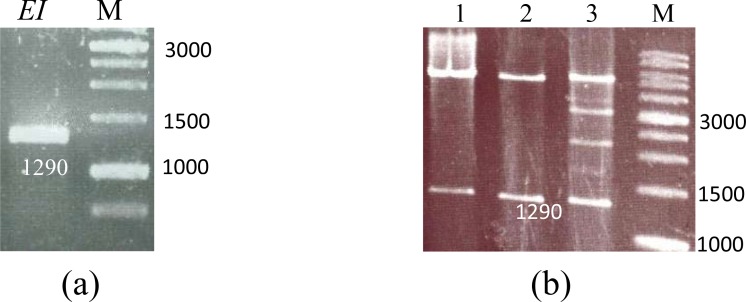
*E. coli* O157:H7 shedding following oral administration in immunized mice.

### Mice challenged with *E. coli* O157:H7

Shedding of bacteria orally administered with 10^10^ CFU of *E. coli* O157:H7 was monitored two weeks after the last immunization in both test and control groups (Fig. 4). The shedding of immunized mice reduced gradually until 10^3^ CFU showing significant decrease compared to that of the control groups (*p* < 0.05).

### Histological examination

The colons and caecums of immunized mice infected with *E. coli* O157:H7 taken on day 8 post infection had normal appearance with well-formed stools and no obvious mucosal thickening. Non immunized infected mice showed visible thickening of the distal colon and diffused stools.

**Fig. 5:**
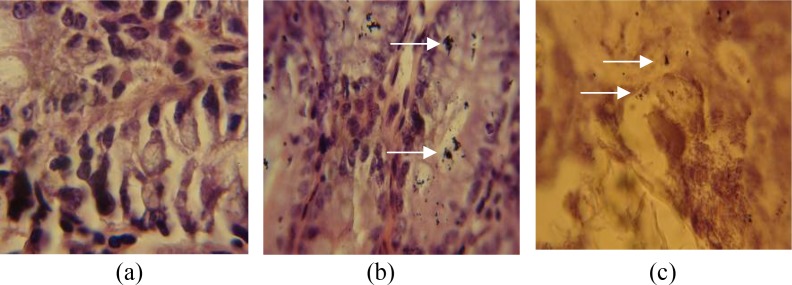
Tissue sampling of colon and cecum from unimmunized and immunized infected mice. A) Untreated mice B), Unimmunized infected mice. C) Immunized infected mice.

The epithelial cells of mice were isolated and the number of residual *E. coli* O157:H7 counted per segment from 3mm of caecum and colon sections. The bacterial counts in immunized mice were approximately 10^3^ in each segment, whereas non-immunized mice infected with *E. coli* O157:H7 showed 10^6^ CFU of bacteria in each segment (*p*<0.05) (Fig. 5).

## DISCUSSION

Vaccination is one of the important options for control of Enterohemorrhagic *Escherichia coli* infection in human and animal (23). A number of studies have shown that proteins encoded by the LEE play key roles in EHEC colonization in the bovine intestine (17, 20, 24). Vaccination of cattle with secreted fractions of EHEC O157:H7 considerably prevented EHEC prevalence and significantly reduced its infection rate (17, 20, 24). A vaccine with highly purified recombinant EspA, induced high-titer antigen-specific IgG1 and salivary IgA. However the antibodies did not protect calves against intestinal colonization by *E. coli* O157:H7 (25). Immunization of cattle with a combination of recombinant EspA, Intimin and Tir have been demonstrated to be protective against *E. coli* O157 challenge (17). In addition, intramuscular immunization of cattle with a vaccine containing recombinant intimin and EspB reduced *E. coli* O157:H7 colonization and shedding (26). A vaccine based on the translocon proteins EspA and EspB and the outer membrane adhesion factor Intimin γ significantly reduced faecal shedding of *E. coli* O157:H7 by orally infected sheeps (27). Finally immunization of mice with a trivalent chimeric protein composed of EspA, Intimin and Tir has been shown to induce strong humoral response and protection against live challenges using EHEC (18). Development of multifactor vaccines is a priority of current vaccine research. Stability of trivalent recombinant proteins is associated with difficulties in its purification for their high weight compared to divalent proteins. Furthermore, separation of divalent proteins by a single linker may favourably impart better folding and stability. With an assumption that these features could effectively enhance the divalent protein immunogenicity, in the present study we hypothesized that a combination of two effective antigens of *E. coli* O157:H7 with a linker separating the domains in a single recombinant protein may work much better than trivalent EHEC antigens (18, 28). In this study, we amplified a divalent recombinant EI protein composed of two EHEC immunogens: EspA lacking 36 amino acids from the N-terminal of the protein (EspA _120_) from type III secretion system (11) and the 282 amino acids from the carboxy-terminal of Intimin (intimin 280) (15, 29). The two fragments were combined with a linker (EAAAK)4. As reported in our previous study (19). EspA filaments act as an organelle that delivers effector proteins into the host cell and hence antibodies that bind to EspA could inhibit assembly of the organelle. On the other hand the antibodies raised against intimin could interfere its interaction with Tir which is considered as effector protein (17). Our finding demonstrated that subcutaneous vaccination of mice with recombinant chimeric protein including EspA 120-linker-Intimin282 can develop high titer of IgG in immunized mice serum and protect against live challenges with EHEC compared to the pervious works carried out with either proteins alone (24, 25). Statistical comparison of this work with previous report (18) in our laboratory showed significant differences between EspA-Intimin and EspA- Intimin-Tir vaccination results. The assay showed that recombinant EI could also prevent epithelial attachment of EHEC O157:H7 and reduces duration of bacterial shedding.

The macroscopic caecum and colon examination of treated mice showed significant difference in the appearance as compared to the control (Fig. 5). There was also a significant difference in the number of bacteria in caecum and colon of immunized and non-immunized mice challenged with *E. coli* O157:H7 (Fig. 5). There was a correlation between these findings and those of shedding studies in stool samples. The results presented in this research indicate that the rEI protein can somewhat protect the mice against *E.coli* O157:H7 as well as the previous studies conducted by other researchers (17, 18, 30).

Inconclusion, the subunit vaccines based on EspA_120_ and Intimin_280_ could be considered as a vaccine candidate against *E. coli* O157:H7. Cattles being the main reservoir are suggested for a field trial.
